# A Retrospective Analysis of Pediatric Cases Handled by the MSF Tele-Expertise System

**DOI:** 10.3389/fpubh.2014.00266

**Published:** 2014-12-08

**Authors:** Daniel Martinez Garcia, Laurent Bonnardot, David Olson, Harriet Roggeveen, Jaap Karsten, Peter Moons, Myrto Schaefer, Joanne Liu, Richard Wootton

**Affiliations:** ^1^Medical Department, Operational Centre Paris, Médecins Sans Frontières, Paris, France; ^2^Fondation Médecins Sans Frontières, Paris, France; ^3^Department of Medical Ethics (EA 4569), Paris Descartes University, Paris, France; ^4^Operational Centre Paris, Médecins Sans Frontières, Paris, France; ^5^Operational Centre Amsterdam, Médecins Sans Frontières, Amsterdam, Netherlands; ^6^Médecins Sans Frontières International President, Geneva, Switzerland; ^7^Norwegian Centre for Integrated Care and Telemedicine, University Hospital of North Norway, Tromsø, Norway; ^8^Faculty of Health Sciences, University of Tromsø, Tromsø, Norway

**Keywords:** low income countries, limited resource settings, telemedicine, telehealth, pediatric, humanitarian, emergency medicine

## Abstract

We conducted a retrospective analysis of all pediatric cases referred by Médecins Sans Frontières (MSF) field doctors via the MSF telemedicine system during a 4-year period from April 2010. A total of 467 pediatric cases were submitted, representing approximately 40% of all telemedicine cases. The median age of the patients was 4 years. The median response time (i.e., the interval between the case being submitted and the first response from a specialist) was 13 h (interquartile range 4–32 h). We selected a random sample of 12 pediatric cases in each of four age categories for detailed analysis by an experienced MSF pediatrician. In the 48 randomly selected cases, the mean rating for the quality of information provided by the referrer was 2.8 (on a scale from 1 = very poor to 5 = very good), and the mean rating for the appropriateness of the response was 3.3 (same scale). More than two-thirds of the responses were considered to be useful to the patient, and approximately three-quarters were considered to be useful to the medical team. The usefulness of the responses tended to be higher for the medical team than for the patient, and there was some evidence that usefulness to both groups was lower in newborns and adolescent patients. The telemedicine system allows the quality of the medical support given to medical teams in the field to be controlled objectively as there is a record of all cases and answers. Telemedicine has an important role in supporting the aims of medical humanitarian organizations such as MSF.

## Introduction

Pediatric cases represent the highest proportion of patients in Médecins Sans Frontières (MSF) programs. Providing rapid and useful support to the teams in the field is at the top of the organization’s priorities. In MSF, most of the clinical work is conducted by general physicians, clinical officers, and nurses with very heterogeneous experience. This may include pediatric inpatient and outpatient care, malnutrition, or tropical medicine in limited resource settings.

Up to 55% of MSF medical activities are in conflict or unstable locations. Furthermore, almost all of MSF activities concentrate in remote or hard to reach areas. (1) This increases the challenge for medical staff and also delays time to consultation. Thus, many patients reach medical facilities with late presentations of diseases. Ensuring access to basic and vital medical services is at the heart of MSF objectives. Most MSF projects provide a range of generalist services in the field focusing on the most urgent needs of vulnerable populations. Some projects focus on a specific disease or medical condition that we consider neglected (e.g., HIV or TB); it is only in this type of project that access to specialized care will be possible routinely. In some settings, security risks are so high that it is very difficult to provide hands-on support to local medical teams. Telemedicine is one possible way in which access to specialist or more appropriate medical consultation might be improved.

In 2010, using a highly secure web-based messaging system, MSF began to pilot two telemedicine networks to support medical field workers. One was operated in French and one in English; a third Spanish network was brought into operation in 2012. In late 2013, the three telemedicine networks were combined into a single multilingual system, telemed.msf.org. A total of 1147 cases (both adult and pediatric) have been submitted through the system, and a survey showed that there was high satisfaction from the users (2).

In the integrated multilingual network, when a case is submitted a case-coordinator reviews the case to decide which specialist(s) will be the best to provide an answer. The coordinator then allocates the case. If an answer is not received within a certain time frame (maximum 24 h), the case is reallocated to another specialist, in order to ensure that the referrer in the field receives an answer in the shortest time possible.

Starting in October 2013, individual case follow-up (i.e., a progress report) was requested automatically from referrers, 21 days after each new case was submitted.

We have conducted a quantitative and qualitative analysis of the pediatric cases submitted through the system.

## Materials and Methods

We conducted a descriptive retrospective analysis of all pediatric cases (age recorded under 18 years) referred by MSF field medical staff to the MSF telemedicine platform from April 2010 to March 2014, inclusive. This represented 467 cases out of 1147 cases in the system. The telemedicine system has a database from which authorized people can retrieve information from specific clinical cases, by selecting specific characteristics such as age. Ethics permission was not required, because patient consent had been obtained prior to submitting each case and the work was a retrospective chart review of anonymized data conducted by the organization’s staff in accordance with its research policies.

### Case characteristics

Demographic and other data were extracted from the database and stored in a spreadsheet for analysis of the case characteristics.

### Detailed review of sample cases

In addition, because it was impractical to conduct a detailed review of the 467 pediatric cases, a random sample of approximately 10% of the cases was reviewed instead. The 48 cases were chosen randomly in four age groups, i.e., 12 pediatric cases were selected by stratified random sampling. The random selection was done using a randomization program according to the case number in the system. The age categories were: birth to 4 weeks, 1 month to 2 years, 2–10 years, and 10–18 years. The cases were assessed by an experienced MSF pediatrician who had both practised as a pediatric expert and as an MSF field referrer. He received the list of selected cases, obtained access to the electronic case record, and used a spreadsheet to summarize his findings as explained below. This reviewer was blinded to the process of selection of the 48 cases.

Three domains were assessed:

1. The *quality of the information provided* by the field doctor was rated on a five-point Likert scale (1 = very poor; 2 = poor; 3 = sufficient; 4 = good; 5 = very good). This rating also took into account the clarity of the request. For example, in some instances, the case had been uploaded for “routine expert advice” such as for an X-ray interpretation, while in others, the referrer had clearly asked specific questions, such as: “What should I do? What is the treatment? What is the diagnosis?”
2. The *appropriateness of the response* given by the specialist was also rated on a five-point Likert scale (1 = very low; 2 = low; 3 = sufficient; 4 = high; 5 = very high). This rating took into consideration whether the response provided was:
  – clear (easy to follow and implement),
  – accurate (medically in accordance with the best medical information available),
  – appropriate for the patient (whether the specialist had considered the patient as a whole, rather than commenting on a particular element, such as an X-ray image alone),
  – appropriate to the context (relative to the capacity of the specialist to understand the resources available in the field, i.e., referral capacity).
3. The *value (usefulness) of the response* was rated as Yes/No. Two perspectives were considered: value to the patient, and value to the medical team. A response that was useful for the patient was one providing helpful information regarding diagnosis, treatment, management, prognosis, and/or the need to transfer the patient. A response that was useful to the doctor was one where an appropriate answer was provided to the question(s) posed in the referral. If the patient died while the answer was being sent, the response was rated as non-useful. In a substantial number of cases, it was difficult to assess the usefulness of the response as there was no feedback documented in the system. In these cases, the usefulness was rated as unknown or undetermined.

### Individual follow-up from the referrer

The progress reports based on closed-ended questions relative to the user’s satisfaction and benefit were reviewed.

## Results

### Case characteristics

**Figure 1:**
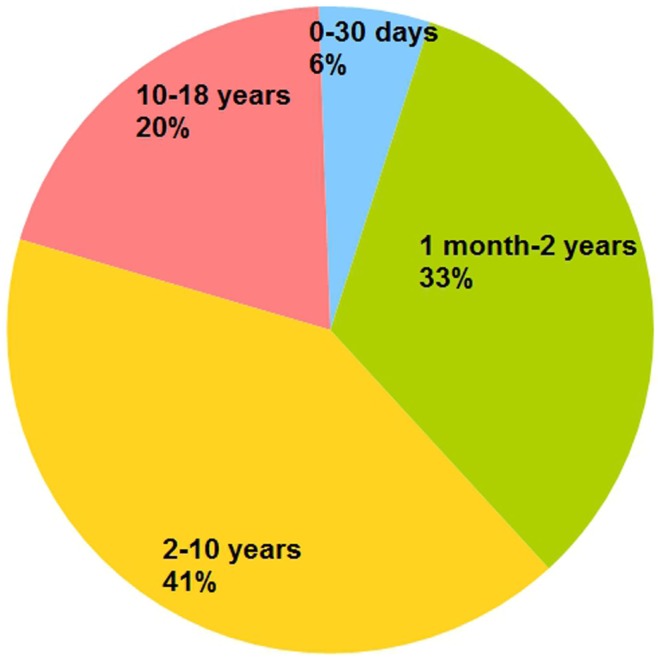
**Age of pediatric patients (*n* = 467)**.

During the study period, a total of 467 pediatric cases were submitted by medical staff from MSF field sites. These pediatric cases comprised 41% of all telemedicine cases. Among the pediatric patients, there were 256 males and 201 females (in 10 cases, the sex of the patient was not recorded). The median age of the pediatric cases was 4 years (interquartile range 1–9 years). The number of patients in the four age categories was: 26 for 0–30 days, 155 for 1 month to 2 years, 193 for 2–10 years, and 93 for 10–18 years (Figure 1). The cases were submitted from 28 countries (Table 1). Half of the cases were submitted from three countries: Central African Republic (23%), South Sudan (13%), and Ethiopia (11%).

**Table 1:** **Countries of origin of cases**.

Country of origin	No. of cases
Afghanistan	6
Bangladesh	4
Cambodia	12
Central African Republic	106
Chad	23
Congo, Republic of Brazzaville	2
Democratic Republic of the Congo (Kinshasa)	27
Ethiopia	50
(France)	17
Guinea	4
Haiti	2
India	2
Kenya	13
Madagascar	4
Malawi	26
Mali	8
Myanmar, Burma	4
(Netherlands)	4
Niger	1
Sierra Leone	2
South Sudan	59
(Spain)	1
Sudan	19
(Switzerland)	7
Tajikistan	47
Uganda	8
Uzbekistan	1
Yemen	8
Total	467

*A small number of cases apparently from industrialized countries (shown in parentheses) were submitted via the MSF headquarters office responsible for a field hospital elsewhere*.

Submission of the cases resulted in 761 queries (a case always results in at least one query being sent to a specialist; if there are requests for a subspecialist opinion, then a single case may result in several queries), a ratio of 1.6 queries per case. The median response time (i.e., the interval between the case being submitted and the first response from a specialist) was 13 h (interquartile range 4–32 h).

**Figure 2:**
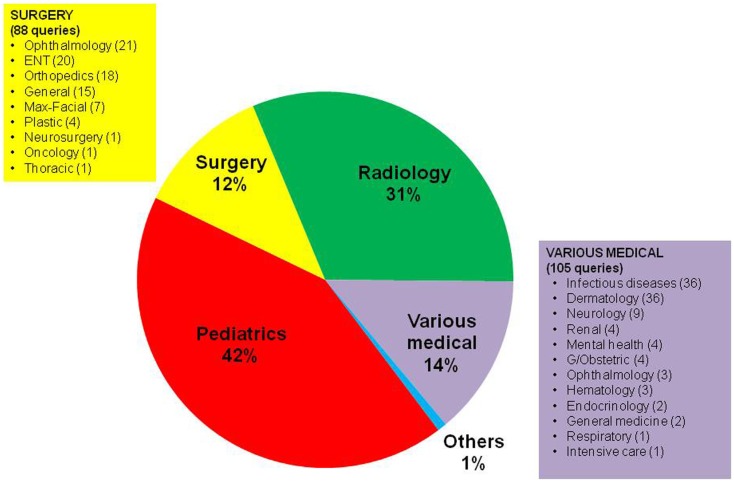
**Types of queries in the pediatric cases, categorized by the specialty of the expert to whom they were sent (*n* = 761)**. “Pediatrics” represents general pediatrics.

The queries covered a wide range of medical and surgical specialties (Figure 2). Among medical subspecialties, the three most common types of referral were for tropical diseases (36), dermatology (36), and neurology (9); among surgical subspecialties, the three most common types of referral were for ophthalmology (21), ENT (20), and orthopedics (18).

Over the 467 cases recorded, the majority (42%) were answered by pediatricians (Figure 2). This is not surprising as MSF pediatric advisers at headquarters are the first line responders for these cases and are often the focal point for centralizing advice from other experts and sub-specialists and thus provide a comprehensive answer to the field.

### Detailed review of sample cases

In the 48 randomly selected cases, the mean rating for the quality of information provided by the referrer was 2.8 (Table 2, range 1–5), and the mean rating for the appropriateness of the response was 3.3 (range 1–5), implying an acceptable/good response given to the field. There was no evidence from the ratings in the subgroups that quality or appropriateness was substantially different across the different age groups of the patients, see Table 2.

**Table 2:** **Assessment of randomly selected cases**.

Age group	No. of cases	Mean quality score^a^	Mean appropriateness score^a^	Useful to patient?	% Useful to patient	Useful to medical team?	% Useful to medical team
0–18 years	48	2.8	3.3	No = 12; yes = 29	71	No = 10; yes = 31	76
0–4 weeks	12	3.3	3.4	No = 5; yes = 6	55	No = 4; yes = 7	64
1 month to 2 years	12	3.1	3.4	No = 2; yes = 10	83	No = 2; yes = 10	83
2–10 years	12	2.0	3.5	No = 0; yes = 8	100	No = 0; yes = 8	100
10–18 years	12	2.6	2.9	No = 5; yes = 5	50	No = 4; yes = 6	60

*^a^Scored from 1 = very poor to 5 = very good*.

*The columns “Useful to patient” and “Useful to medical team” contain some missing data (7 cases of the 48), where usefulness could not be determined*.

**Figure 3:**
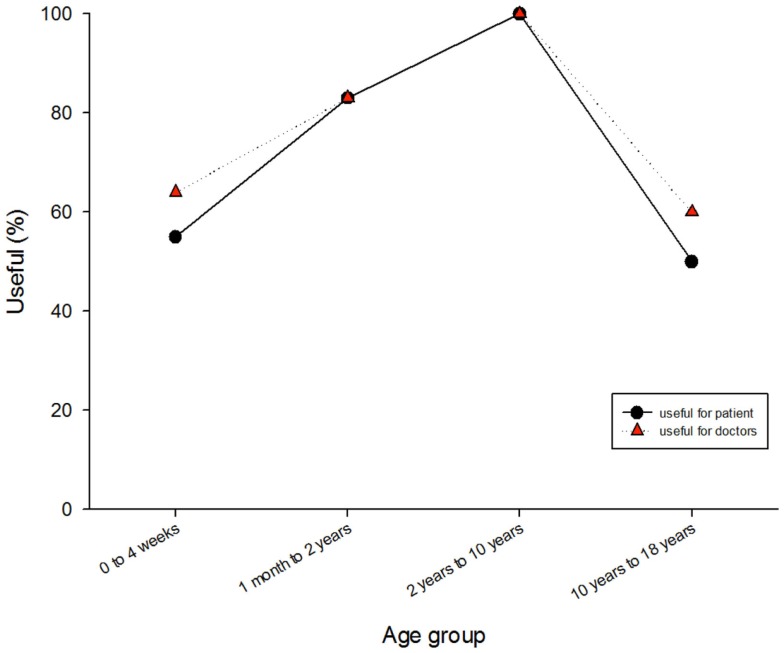
**Usefulness of the responses to the patients and to the medical team**.

Approximately two-thirds of the responses were considered to be useful to the patient, and approximately three-quarters were considered to be useful to the medical team. The usefulness of the responses tended to be higher for the medical team than for the patient, and there was some evidence that usefulness to both parties was lower for the newborns and the adolescent patients (Figure 3).

### Individual follow-up from the referrer

In the period October 2013 to March 2014 inclusive, 42 requests for follow-up were issued. A total of seven progress reports were provided (response rate of 17%). The responders were generally positive about the value of the teleconsultation (Table 3).

**Table 3:** **Summary of the progress report data provided by referrers of pediatric cases**.

	Do not know	No	Perhaps	Yes	Percentage Yes
(1) Was the case sent to an appropriate expert?	1			6	86
(2) Was the answer provided sufficiently quickly?		1		6	86
(3) Was the answer well adapted for your local environment?		3		4	57
(4) Were you able to follow the advice given?		1		6	86
(6) Did you find the advice helpful?				7	100
(7) If Yes, did it (tick any that apply)					
– Clarify your diagnosis		1		5	83
– Assist with your management of the patient				5	100
– Improve the patient’s symptoms		4		1	20
– Improve function		5			0
(8) Do you think the eventual outcome for the patient will be beneficial for the patient?			5	2	29
(9) Was there any educational benefit to you in the reply?				6	100
(10) Was there any cost-saving as a result of this consultation?		5		2	29

*Free-text comments*.

* The outcome cannot be evaluated fully because the patient defaulted after the last necrosis was debrided, at least since the change of antibiotics there was no newly formed necrosis*.

* Patient follow-up has been lost*.

* Even if treatment options are limited at our level, advice on this difficult cases is very helpful to orient diagnosis and give patient proper advice*.

* The service is EXCELLENT, always well adapted to our environment, understanding of our limitations, and sometimes our lack of professionalism! Answers are always very rapid and extremely useful to the field and consequently the patients. We could not manage without them!*.

* Excellent service*.

* Telemedicine is appreciated a lot!*.

## Discussion

We conducted a retrospective analysis of all pediatric cases referred by MSF field doctors via the MSF telemedicine system during a 4-year period.

### Review of the literature

The majority of previous work on telepediatrics has been video-based, and has concerned high income countries. For example, much early work was done in Queensland (Australia) approximately 15 years ago (3, 4), and subsequently in the United States (US) (5). However, video links are expensive and the necessary bandwidth is not always available in low-resource settings. There has been little video-based work in low income countries: a pilot service in India (6) and the MSF Somalia project (7) are rare examples. The latter study reported that 346 cases (9% of the total) were referred for telemedicine and in 222 children (64%), “*a significant change was made to initial case management, while in 88 (25%), a life-threatening condition was detected that had been initially missed*.” Internet video has been used for surgical planning in some low and middle income countries (LMIC) prior to visits by US surgical teams (8, 9), but this did not specifically target the pediatric age group.

There has been a pediatric component to cases managed by store-and-forward telemedicine in other networks (e.g., US Pacific, Swinfen), but no specific reporting of the experience in the literature. Some pediatric case reports have been provided. (10) Email has been used for pediatric orthopedics in Djibouti. (11) As far as we are aware, there are few other reports concerning store-and-forward telemedicine for pediatric work in low-resource settings.

### Characteristic of MSF pediatric field work

In the present study, there were a large number of X-rays. This was mainly due to the over-representation of HIV and TB projects using the system. Overall, more than 40% of cases submitted through the MSF telemedicine system involved patients under the age of 18 years. This is in line with the normal pyramid of ages in developing countries, where we would expect about 40–50% of the population to be less than 18 years of age.

The usefulness of the answers produced via the telemedicine system appeared to be lower for newborns and for adolescent patients (Figure 3). These two groups have features that require more knowledge and experience to deal with. Adolescents are a difficult group to reach and they often present with diseases at a more advanced stage. The newborn group is marked by congenital problems that are hard to diagnose without appropriate medical technologies in the field and which require highly specialized management. Most of the neonatal cases were advised by non-neonatologist specialists.

The MSF telemedicine system was initially designed to be used for complex cases (excluding life-threatening emergencies). However, it was often used for “non-complicated” cases for which protocols are available in MSF guidelines that could have been easily applied. (12) It is a fact that most MSF field work including pediatric clinical work is performed by general physicians, clinical officers, nurses, or midwives. The case content analysis revealed that in some cases the field medical teams did not even have a suspected diagnosis (working hypothesis). This might indicate the lack of familiarity of field workers with basic pediatric principles and procedures, as well as a lack of use or appropriateness of existing tools such as guidelines. The role of the headquarters pediatric advisors is thus crucial to ensure that field teams do not overlook essential elements, physical findings, or specific information that would help them to manage cases by themselves. This also demonstrates the complexity of field management of pediatric cases and the growing need for having expert support for adapting protocols to the field environment.

### Strengths of the system

The review of the MSF tele-expertise system demonstrates some strengths. These include:

1. it represents a secure, reliable, and efficient method of obtaining rapid answers for difficult cases (the median response time was only 13 h);
2. there were no concerns about data confidentiality. On the other hand, MSF also uses email to provide pediatric support for its field workers, although email is not a secure way to communicate and should not be used – especially in sensitive contexts – to transmit any identifiable patient data (e.g., HIV patient status, victims of violence, ethnic tensions);
3. data can be retrieved easily and analyzed for quantitative or qualitative analysis. The system also provides an audit trail in case of any future enquiries;
4. it allows multidisciplinary/interspecialty management under pediatric advisor supervision and control (gathering sub-specialty advice);
5. useful pediatric documents, protocols and guidelines are easily accessible through the platform and available to users;
6. having a coordinator available online around the clock guarantees the rigorous follow-up of each case, especially during periods where an overbooked pediatric advisor is not available (due to time off, sick leave, weekend, night time).

### Limitations of the study

The present study only reports on clinical cases submitted through the telemedicine system. Thus, it does not provide a comprehensive picture of all MSF pediatric clinical cases. This is because a substantial proportion of cases requiring support from MSF pediatric advisors are sent from the field via other means, such as email, Skype, SMS, telephone, or even through social media platforms. For security and other reasons, MSF is trying to reduce the communication of clinical cases outside the telemedicine platform.

For the purpose of the present work, we only reviewed information available within the telemedicine system. As shown in Table 1, the use of the telemedicine system varied heavily, depending on the country and project acceptance and understanding of the system. The number of cases sent by a project was therefore a reflection of the acceptance of the telemedicine system in that particular country. Future comparisons will show the trends from each project and we will be able to assess acceptance and efficacy of the system.

A second limitation is that a retrospective analysis was carried out, with no control arm for a comparator. Moreover, the availability of only limited feedback about outcomes of cases may represent a source of bias. Although the preliminary feedback from referrers suggests that they find the system useful (Table 3), a systematic survey was not conducted. On the other hand, a much larger previous survey of users also found that their overall opinion was positive (2).

Finally, the qualitative assessment was done by a single pediatrician and therefore the results must be interpreted with caution. A future study would be strengthened if it used a panel of observers. Part of the validation of any future methodology would involve developing measures in which there was good agreement between and within observers.

### Perspectives

Despite the strengths of the telemedicine system, it also has weak points. This leads us to make the following recommendations:

1. Feedback to the specialist should be mandatory, not only to keep the experts motivated but also for quality improvement purposes.
2. The quality of pictures (X-ray, photographs, ultrasound scans) was sometimes poor. This is a well-known problem in store-and-forward telemedicine (13) and technical information should be given to the field users with the aim of improving image quality.
3. A pediatric standard referral form might be useful to allow the teams in the field to provide case information in a more systematic and organized way.
4. Since pediatric cases represent a substantial proportion of all telemedicine cases, having a specific pediatric case coordinator with knowledge of the pediatric expert network might improve the efficiency and quality of the system.
5. There was some delay in the allocation process for a small number of cases, leading to a slight delay in obtaining the final answer for the field. This problem could be addressed by having a clear pattern of allocation (i.e., list of experts in first, second, and third line for each MSF operational section).
6. Efforts should be made to obtain follow-up data for all cases.
7. Briefing all medical staff going to the field about the telemedicine system should be mandatory to increase its use and reduce the use of parallel and non-secure platforms for clinical case discussion between the field and the medical department.

### Future developments

In the near future, with better reliability of new technology including mobile devices and a broader access to the Internet, we envisage that the telemedicine system will provide more direct support (e.g., at the bedside) to more field doctors. A telemedicine application for mobile devices would allow users to create their referral offline at the patient’s bedside and then have it sent automatically as soon as an Internet connection was established. Real time telemedicine with live chat or real time video could also allow the telemedicine system to provide support for life-threatening emergencies, or for cases requiring very rapid decisions from the medical teams in the field. In order to be able to provide this new real time service, a pool of online experts would be required. A large telemedicine center, probably a virtual center, could be created to coordinate and respond to multiple cases being received.

## Conclusion

Given the significance of pediatric cases in the daily activities of MSF and the impossibility of having a trained pediatrician present at all field sites where children receive care, means that access to remote advice is important. A telemedicine system can greatly improve the level of medical care provided to patients and reduces the isolation of field doctors in their practice. Telemedicine is also valuable in insecure, unstable settings where the number of medical personnel needs to be minimized out of concern for staff safety.

Confidentiality and security of communication regarding patient information provided through telemedicine should lead healthcare organizations to consider using this as their sole method of communication with the field with regards to patient information. However, this will require easier access for field workers to the system, e.g., via mobile devices, and appropriate arrangements at headquarters level to manage the workload appropriately.

Medical humanitarian organizations such as MSF work to reduce the health gaps for the most vulnerable populations in the most difficult contexts. Telemedicine has an important role in supporting those aims.

## Conflict of Interest Statement

The authors declare that the research was conducted in the absence of any commercial or financial relationships that could be construed as a potential conflict of interest.
